# Lipids in major depressive disorder: new kids on the block or old friends revisited?

**DOI:** 10.3389/fpsyt.2023.1213011

**Published:** 2023-08-17

**Authors:** Anna Rosa van der Heijden, Tom Houben

**Affiliations:** Department of Genetics and Cell Biology, School of Nutrition and Translational Research in Metabolism (NUTRIM), Maastricht University, Maastricht, Netherlands

**Keywords:** major depressive disorders, sphingolipids, cholesterol, fatty acids, obesity, type 2 diabetes

## Abstract

Major depressive disorder (MDD) is a psychiatric mood disorder that results in substantial functional impairment and is characterized by symptoms such as depressed mood, diminished interest, impaired cognitive function, and vegetative symptoms such as disturbed sleep. Although the exact etiology of MDD is unclear, several underlying mechanisms (disturbances in immune response and/or stress response) have been associated with its development, with no single mechanism able to account for all aspects of the disorder. Currently, about 1 in 3 patients are resistant to current antidepressant therapies. Providing an alternative perspective on MDD could therefore pave the way for new, unexplored diagnostic and therapeutic solutions. The central nervous system harbors an enormous pool of lipids and lipid intermediates that have been linked to a plethora of its physiological functions. The aim of this review is therefore to provide an overview of the implications of lipids in MDD and highlight certain MDD-related underlying mechanisms that involve lipids and/or their intermediates. Furthermore, we will also focus on the bidirectional relationship between MDD and the lipid-related disorders obesity and type 2 diabetes.

## 1. Introduction

Major depressive disorder (MDD) is a psychiatric mood disorder characterized by a range of psychopathological symptoms that lead to a syndrome causing significant functional impairment. The current Diagnostic and Statistical Manual of Mental Disorders (DSM V) categorizes MDD into 256 symptom presentations, which are organized into “specifiers” to highlight the disorder’s heterogeneity and clinical challenges (1–3). However, the core symptoms specifically associated with MDD are a depressed mood and a general loss of interest, known as anhedonia (1, 2). The lifetime risk of developing MDD is estimated to be 18% (4), but this has increased significantly following the outbreak of the COVID-19 pandemic (5). MDD is also associated with a heightened risk of suicide (6) and other non-communicable diseases (7, 8), leading to increased urgency and awareness of this condition in recent years. According to the Global Burden of Diseases, Injuries, and Risk Factors Study (GBD) 2019, depressive disorders (including MDD) are the most disabling mental conditions and rank among the top 15 primary causes of disease burden globally (9). Future projections suggest that MDD will become the most significant cause of disease burden by 2030 (2).

The heterogeneous nature of MDD (at times even in the same patient) implies the involvement of a plethora of pathophysiological mechanisms. As of today, aberrations in a range of physiological mechanisms have been proposed to contribute to the development of MDD. The most thoroughly researched mechanism revolves around the monoamine hypothesis, stating that a deficiency in the monoamine neurotransmitters norepinephrine and/or serotonin in the brain form the underlying biological basis for the development of MDD (10). The generation of this hypothesis has resulted in the development of many safe and effective antidepressant agents and has, without a doubt, led to unprecedented progress in the field of MDD (10, 11). However, the monoamine hypothesis does not explain observations related to the delay of symptom improvement when monoamine levels in the brain have been restored by antidepressants (12, 13), why antidepressants can reverse anxiety disorders (14) and why drugs that reduce (and not increase) the level of serotonin in the synaptic cleft have an antidepressant effect (15). Another dysregulated physiological mechanism that more recently gained awareness as biological cause for MDD is inflammation [reviewed in detail by others (16–18)]. Apart from the observed increased peripheral cytokine levels in MDD patients (19), other evidences that support an incontrovertible role for the immune system in depression concern the observation that patients suffering from immune disorders (from autoimmune or infectious nature) are increasingly prone to develop depressive symptoms (20) and that therapeutic administration of cytokines induces depression (21). A third biological mechanism that has been associated with MDD development is the dysregulation of stress responses via the hypothalamic-pituitary-adrenal (HPA) axis which often manifest itself as a combination of excessive stress-related cortisol release and impaired glucocorticoid receptor-mediated feedback inhibition (22, 23). Despite consistent insights concerning the involvement of the axis in MDD, attempts to translate these findings into clinical therapeutic applications have so far been unsuccessful (24). Next, although the precise biological mechanisms underlying its contribution to behavior are yet to be fully elucidated, neurogenesis has been implicated in the context of MDD (25–27). Neurogenesis refers to the ongoing generation of neurons within the central nervous system (CNS) throughout an organism’s lifespan. Specifically, the subgranular zone (SGZ) of the dentate gyrus in the hippocampus has been identified as the principal region involved in this process (28). Other underlying mechanisms inducing MDD that have gained increasing attention that will not be discussed in further detail here involve glutamate receptor signaling (29) and genetic and/or epigenetic variations (30). Thus, a variety of mechanisms play a role in the development of MDD.

However, considering the central role of the brain in the development of MDD, it is rather surprising that relatively few attention (in comparison to the previously mentioned mechanisms) has been attributed in this field to one of the largest components of the brain: lipids and their intermediates. As an organ, the brain has the second highest lipid content [around 50% dry weight (31)] and carries a plethora of lipid subtypes that have been associated with physiological (32, 33) and neurodegenerative processes (34, 35). In line, in the last two decades, an increasing number of observations have described changes in lipid levels and their metabolism in MDD patients’ circulation and central nervous system. While there is no evidence that lipid changes contribute more to MDD than other known mechanisms, it is becoming clear that lipids influence MDD via known (i.e., by influencing the mechanisms mentioned above) and/or unknown mechanisms. Moreover, depressive symptoms in lipid-related disorders have been associated with more adverse clinical profiles, adding fuel to the argument of a reciprocal relationship between depression and these lipid-related disorders (36, 37). Considering these evidences, this review attempts to provide a short overview of the association between various types of lipids and MDD, as well as to elaborate on specific (potential) underlying mechanisms. Finally, the role of depression in lipid-related disorders will also be reviewed as it adds fuel to the hypothesis of lipids in MDD.

## 2. Systemic and neurological lipid changes in major depressive disorder

### 2.1. Sphingolipids

Sphingolipids are a major class of bioactive lipids whose subcellular location determines their specific function. In short, the basic structure of sphingolipids is composed of a sphingoid base and an amino-linked fatty acid, also referred to as a ceramide. Enzymatic reactions can concomitantly introduce variations to this basic structure, creating a universe of sphingolipids (i.e., the sphingolipidome) including sphingomyelin, ceramide, glycosphingolipids and sphingosine (among many others) (38). Mechanistically, sphingolipids have been linked to key physiological functions such as growth regulation, adhesion, apoptosis and inflammatory responses (38). Moreover, bioactive sphingolipids appear to be implicated in key process in neurodevelopment (39), neuroinflammation (40), systemic inflammation (41) and metabolism (42, 43). For a complete overview of sphingolipid metabolism and functions, we direct the reader to recent reviews that provide an excellent overview of this topic (38, 41).

Considering their ubiquitous expression and large impact on physiological functions, it is axiomatic that sphingolipids have also been linked to MDD. One of the clearest evidences that confirmed this link was the pre-clinical identification of six pharmacological antidepressants (doxepine, fluoxetine, maprotiline, nortriptyline, paroxetine and sertraline) to inhibit the enzyme acid sphingomyelinase (ASM) (44, 45). Indeed, already since 1991, it is known that tricyclic antidepressants functionally inhibit ASM in human post-mortem brains (46). As ASM is responsible for the conversion of sphingomyelin to phosphorylcholine and ceramide, these findings imply an association of sphingomyelin and/or ceramide with MDD pathogenesis. In line with this hypothesis, sphingomyelins were detected as one of six metabolic pathways to prospectively identify female and male MDD patients at risk of future recurrence (47). Furthermore, serum ceramide levels appear to be consistently elevated in human MDD, especially in male patients (48, 49). At the other hand, ceramides were not elevated in the prospective study for recurrent MDD (47), suggesting that circulating ceramide levels might rather be an indicator of MDD severity. Also, increased ceramide levels in the circulation and hippocampal region of rodents have been linked to depression development and severity and its reduction could reverse depressive symptoms (50–52). Indeed, reversal of stress-induced MDD was achieved in mice by blocking ceramide function with intravenous anti-ceramide antibodies or neutral ceramidase. This causal relation was further substantiated by delineating a new underlying pathway by which ceramide inhibits phospholipase D in endothelial cells of the hippocampus, thereby reducing hippocampal phosphatidic acid (52). This finding is in line with previous studies that demonstrated that hippocampal endothelial cells play an essential role in the regulation of neurogenesis during MDD both in preclinical and clinical studies (53, 54). The identification of this novel pathway implies that blood ceramide, along with phospholipase D and phosphatidic acid in the hippocampus, hold promise as potential therapeutic targets for the treatment of MDD. These findings have been substantiated by follow-up studies, providing further evidence for their potential in addressing MDD through therapeutic interventions (55, 56).

**Figure 1:**
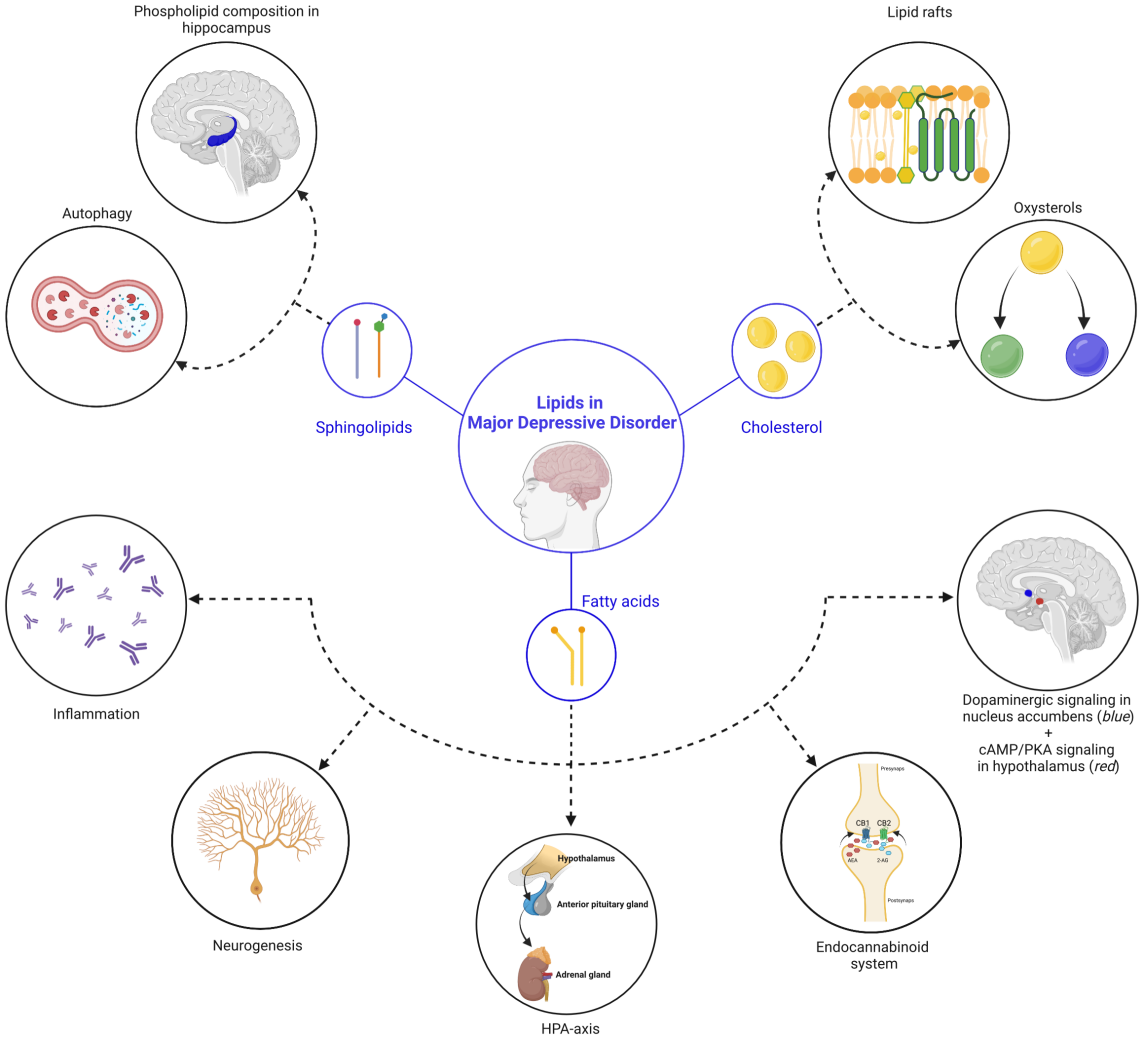
Lipids in major depressive disorder. In this review, we discussed three main classes of lipids (sphingolipids, cholesterol and fatty acids), which are central in processes related to MDD. (*Top left*) Sphingolipid-related mechanisms: (1) the sphingolipid ceramide inhibits phospholipase D in endothelial cells of the hippocampus, leading to a reduction of phosphatidic acid (=a phospholipid) in the hippocampus and deterioration of depressive symptoms. (2) Reversal of depressive symptoms can be achieved via the induction of autophagy in hippocampal neurons, facilitated by the accumulation of sphingomyelin in lysosomes and Golgi membranes, as well as ceramide in the endoplasmic reticulum. (*Top right*) Cholesterol-related mechanisms: (1) the effectivity and responsiveness of antidepressants is related to their ability to accumulate in lipid rafts, a structure that is regulated by cholesterol. Changes in the membrane cholesterol content therefore influence depressive behaviors. (2) Different types of oxysterols (24S-hydroxycholesterol and 25-hydroxycholesterol) were shown to directly influence depressive-related processes. (*Below*) Fatty acid-related mechanisms: (*from left to right*) (1) elevation of lipoxygenase and cytochrome P450 enzymes by polyunsaturated fatty acids leads to a reduction in inflammation and a decrease in the severity of depressive symptoms. (2) While direct influence of fatty acids on neurogenesis is still under debate, the impact of the endocannabinoid (eCB) system on neurogenesis is well-established (also see point 4). (3) Polyunsaturated fatty acids influence the hypothalamic-pituitary-adrenal (HPA) axis, which is a key pathophysiological mechanism of MDD. (4) Endocannabinoids ligands are fatty acids that are linked to MDD. (5) Polyunsaturated fatty acids influence the dopamine system in the nucleus accumbens (*indicated in blue*) during gestation, influencing motivational behavior during adulthood and (6) palmitic acid, a saturated fatty acid, accumulates in the hypothalamus (*indicated in red*) after a high-fat diet and associates with the onset of depression. Created with BioRender.com, endocannabinoid receptor 1, CB1; endocannabinoid receptor 2, CB2; *N*-arachidonoylethanolamine, AEA; 2-arachidonoylglycerol, 2-AG.

Another underlying mechanism that links sphingolipids to MDD pathogenesis is autophagy, which is a self-degradative process essential to maintain energy [the basic process of autophagy is also reviewed here (57, 58)]. Specifically, in preclinical models, the antidepressants amitriptyline and fluoxetine were demonstrated to induce autophagy in hippocampal neurons via a slow accumulation of sphingomyelin in lysosomes and Golgi membranes and of ceramide in endoplasmic reticulum (59). Moreover, a similar effect on autophagy and MDD reversal was also achieved by rapid accumulation of ceramide in the endoplasmic reticulum of the hippocampus via direct inhibition of sphingomyelin synthases, raising sphingomyelin synthase inhibitors as potential new antidepressant agents (59) (also see Figure 1).

Together, a growing number of observations have linked sphingolipid metabolism to MDD development and pathophysiology, opening up several new avenues for pharmaceutical intervention. Yet, the highly regulated nature of the sphingolipidome restricts the use of therapeutic regimens since every minor change in this system might have unexpected consequences. Still, the study of bioactive sphingolipids as possible future antidepressants is valid, although more extensive clinical research is required before clinical implementation can be supported.

### 2.2. Cholesterol

As an essential lipid that is biosynthesized by every mammalian cell, cholesterol is, arguably, one of the most intriguing lipids (60). At a cellular level, cholesterol predominantly localizes in the plasma membrane where it often aggregates with sphingolipids and glycosylphosphatidylinositol-anchored proteins, forming so-called lipid rafts which transduce signaling cascades and regulate membrane trafficking (61). Moreover, cholesterol plays a crucial role in maintaining the structure of the lipid raft as well as its fluidity (62). At a systemic level, lipoproteins [mainly in form of low-density lipoproteins (LDL), very-low-density lipoproteins (VLDL) and high-density lipoproteins (HDL)] enable the hydrophobic cholesterol to be transported to other tissues, ensuring that an increased supply of cholesterol is guaranteed to those locations that would require such demand. However, the central nervous system (CNS) has no access to this cholesterol distribution system, being completely self-reliant in its cholesterol need (32). As cholesterol is indispensable for neurons (63), astrocytes (64) and microglia (65), these CNS cells synthesize cholesterol *in situ* via the Bloch or Kandutsch-Russel pathway [also see (32)]. Finally, cholesterol can be transformed into oxysterols via enzymatic and non-enzymatic reactions, which have key regulatory functions both in the CNS (66, 67) and systemic metabolism (68, 69). For additional information on systemic and CNS cholesterol metabolism, we would like to refer the reader to other reviews (32, 60).

One of the initial associations between MDD and cholesterol refers to the observation that MDD patients demonstrate lower levels of total plasma cholesterol (70–72). However, this claim has been contested by multiple other studies that were unable to replicate these findings (73–75), sometimes even showing opposite correlations (76–79). These inconsistent findings prompted researchers to investigate cholesterol in subgroups of MDD patients.

As mentioned earlier, specifiers are employed to refine and support clinical practice in defining depressive episodes, providing information on the pattern, clinical features, severity, time of onset, and remission status associated with the depressive episode. These specifiers play a crucial role in characterizing the different subtypes of MDD. For instance, the specifier indicating melancholic features, characterized by diminished reactivity of affect and mood, a pervasive and distinct quality of depressed mood that worsens in the morning, along with anhedonia, guilt and psychomotor disturbance signifies a melancholic subtype (2). In contrast, in cases of psychotic MDD, patients experience the co-occurrence of a depressed mood and psychosis. The psychosis often presents in the form of nihilistic delusions, characterized by the belief that negative events or outcomes are imminent (80). It is also worth noting that psychotic MDD patients are more likely to suffer from treatment-resistant depression (81). Finally, another common specifier concerns atypical MDD, which describes patients that show mood reactivity and two or more of the following four features: hypersomnia, leaden paralysis, increase in appetite (or significant weight gain), a long-standing pattern of interpersonal rejection sensitivity that results in significant social or occupational impairment (82).

By focusing on these specific subgroups of MDD, researchers have been able to shed an alternative light on the complex relationship between MDD and cholesterol: (1) atypical depression in patients positively associates with obesity-related parameters such as increased BMI, waist circumference, fat mass (83, 84) and higher total and LDL cholesterol (76); (2) melancholic MDD patients rather associate with weight loss (85) and with lower levels of HDL cholesterol (76) and (3) psychotic MDD patients demonstrate increased serum total cholesterol and LDL cholesterol levels, but lower HDL cholesterol levels (86). Additionally, initial associations of the cholesterol precursors desmosterol and 7-dehydrocholesterol with MDD diagnosis in patients appeared to be confounded by the use of trazodone, a psychotropic medication (87). Accounting for medication use and stratification of MDD patients into subtypes [f.e. based on the specifiers described by the DSM V (3)] therefore increases the likelihood of providing more consistent cholesterol-related associations to MDD that were previously unknown, inciting researchers to further investigate the exact function of cholesterol in the respective MDD subtypes.

However, elaborative mechanistic research investigating the causal role of cholesterol in MDD is relatively scarce as compared to its well-researched clinical associations. Still, there are direct and indirect evidences to claim that cholesterol plays a role in MDD. Low-density lipoprotein receptor knockout (*Ldlr*^−/−^) mice, which are characterized by hypercholesterolemia, display depressive-like behavior (88). In the context of antidepressant sensitivity, cholesterol-lowering medication are currently in clinical trials as augmentation therapy for treatment-resistant depression (TRD) (89), though recent observations indicated no added effect of simvastatin on TRD (90). At a cellular level, the role of cholesterol in antidepressant response has been linked to lipid rafts in preclinical models (91). Specifically, by accumulating in lipid rafts, antidepressants mediate the extrusion of the heterotrimeric G protein Gsalpha (Gsα), which relates to antidepressant effects (92) and response (93). These findings raise the question whether modulation of membrane cholesterol could influence antidepressant effects? In line with this proposition, 27 patents have reported benefits of complexing antidepressant drugs with cyclodextrins (known to influence membrane cholesterol content), though underlying mechanisms were not described (94). Therefore, whether the described improvements of cyclodextrins on antidepressant efficacy are related to changes in membrane cholesterol content has to be further investigated in the future. Regardless, considering that antidepressant treatment was shown to induce plasma cholesterol changes (95), which also significantly influenced treatment response (96, 97), these myriad of observations, to the least, suggest an involvement of cholesterol in antidepressant response.

Finally, due to their regulatory role in brain cholesterol turnover (98, 99), certain oxysterols have been linked to MDD-related processes. The oxysterol 24S-hydroxycholesterol (24S-HC) is generated via enzymatic oxidation of cholesterol by the enzyme cytochrome P450 family 46 subfamily A member 1 (CYP46A1) which occurs in human neurons (98). Further, 24S-HC is a positive allosteric modulator of N-methyl-D-aspartate receptors (NMDARs), which mediate neurotransmission signals throughout the CNS related to learning, memory and mood (100). Strikingly, preclinical models showed that 24S-HC acts mainly on the GluN2B subunit of NMDARs (101, 102), which is also the main target for the antidepressant ketamine (103). At the other hand, another oxysterol, 25-hydroxycholesterol (25-HC), displayed antagonistic effects on 24S-HCs ability to modulate NMDARs preclinically, supporting a regulatory role for oxysterols in NMDAR modulation (104). As the antidepressant effect of ketamine is mediated via NMDAR, it is acceptable to assume that changes and/or aberrations in oxysterol metabolism influence mood-related processes. In line, while plasma levels of 24S-HC did not differ between depressed and healthy patients (105), its prefrontal cortex levels were increased in patients who committed suicide (106) (also see Figure 1).

Overall, while many studies have investigated plasma cholesterol in MDD cohorts, our impression is that the main evidence for the role of cholesterol in MDD manifests itself predominantly in the form of pharmacological antidepressant-related research. As these pharmacological studies imply an involvement of cholesterol in mood-related processes, correcting for medication use and stratification of MDD patients into subgroups could enhance our understanding of cholesterol (and its precursors) in MDD cohort studies.

### 2.3. Fatty acids

Finally, considering their abundance in the human body, the last lipid type that we will cover in this review concerns fatty acids. Fatty acids are generally divided into saturated and unsaturated fatty acids, the latter referring to the presence of double bonds in the carbon chain (107). Both types of fatty acids are abundantly present in the brain, where they are primarily esterified to the phospholipid cell membrane, coordinating the function and structure of neurons and glial cells (108). Besides structural support, fatty acids are involved in numerous neurological signaling pathways including cell survival, neurogenesis, synaptic function and brain inflammation. Further, while saturated fatty acids can be synthesized *de novo* by the brain, polyunsaturated fatty acids (PUFAs) are supplied via the diet (108). More information on (poly)unsaturated and saturated fatty acids is described in other excellent manuscripts as this is beyond the scope of the current review (108–110). Finally, a major fatty acid-related system that deserves specific attention is the endocannabinoid (eCB) system. The two best studied eCB ligands, *N*-arachidonoylethanolamine (AEA) and 2-arachidonoylglycerol (2-AG), are arachidonic acid derivatives which is a n-6 polyunsaturated fatty acid (111). Other essential components that are involved in the eCB signaling system include (1) a minimum of two G-protein-coupled receptors (GPCRs) known as cannabinoid type-1 and type-2 receptors (CB1R and CB2R) and (2) synthetic and degradative enzymes as well as transporters that regulate the levels and actions of eCBs at the receptors (112) for more in depth reviews on the eCB system, we refer the reader to (113, 114).

Compared to cholesterol and sphingolipids, fatty acids, and in particular PUFAs, have been extensively investigated in the context of MDD as clinical and preclinical studies have suggested a role for PUFAs in regulating mood. Specifically, levels of the n-3 PUFAs eicosapentaenoic acid (EPA) and docosahexaenoic acid (DHA) were reduced in the blood of depressive patients compared to healthy subjects, while no differences in n-6 PUFAs were observed (115). More evidence for this link comes from brain studies showing reduced DHA and EPA levels in the brain of MDD patients (116–118). At the other hand, PUFAs did not associate with the prospective risk of MDD recurrence (119), implying that the link between PUFAs and mood appears to be solely restricted to initial MDD diagnosis. Incontrovertible evidence for the impact of PUFAs on MDD comes from cross-national epidemiological surveys observing an inverse correlation between *per capita* fish or seafood (which is high in PUFAs) consumption and lifetime prevalence rate of MDD (120, 121). Moreover, shifting away from a fish-based diet towards a Western-type diet increased rates of depression, seasonal affective disorder, anxiety and suicide (122) and an observational study even suggested that a diet rich in n-3 PUFAs decreases the risk in elderly individuals (116). These dietary findings could also be replicated in animal studies showing that reduced n-3 PUFA intake leads to depressive-like behavior in rats, mice and monkeys (123–126). For a more detailed overview of the association studies between PUFAs and MDD (108, 127, 128).

Several mechanisms have been implicated in mediating the beneficial effects of n-3 PUFAs, some of which are related to already known pathophysiological mechanisms related to depression. Firstly, PUFAs are known anti-inflammatory agents (129, 130). By increasing lipoxygenase (LOX) and cytochrome P450 (CYP450), EPA and DHA were shown to reduce inflammation in a hippocampal progenitor cell line. Strikingly, these findings could be replicated in a clinical study of 22 MDD patients, demonstrating associations between LOX and CYP450 metabolites and depressive symptom severity (131). Building further on this idea, pro-resolving lipid mediators (SPMs) could mediate the reduction of inflammation that is induced by the increase in PUFAs (132). In short, SPMs are lipid mediators that are mainly derived from the conversion of n-3 fatty acids EPA, DHA and docosapentaenoic acid (DPA) via cyclooxygenase and LOX and are known to mediate the resolution of inflammation (133, 134). In line, EPA supplementation to MDD patients resulted in improved symptoms of depression, reduced systemic inflammation and increased plasma concentrations of SPMs (132), highlighting the resolution of inflammation via n-3 PUFA-induced increase of SPMs as a potential new approach for MDD.

Besides inflammation, PUFAs have also been directly linked to the elevation of neurogenesis, though confirmation in clinical studies is, to our knowledge, lacking at this point. Third, circumstantial evidence also suggests a link between the MDD effect of PUFAs and the HPA-axis as chronic EPA supplementation prevents increases in plasma corticosterone levels in mice (135, 136). Moreover, lower plasma n-3 PUFA levels were associated with an hyperactive HPA-axis in the general population (137) and plasma DHA levels negatively associated with evening cortisol concentrations in recurrent MDD patients, adding fuel the argument that PUFAs might play a role in HPA-axis activity (138). Further, another underlying PUFA-influenced mechanism that was recently identified relates to the dopamine system in the brain. Specifically, via a n-3 PUFA deficiency during gestation, the authors identified changes in brain phospholipid content which eventually manifested itself into motivational deficits in adulthood. This finding was further associated with increased inhibition of D2 receptor-expressing medium spiny neurons onto dopamine D1 receptor-expressing neurons in the nucleus accumbens (139). Though less research has been conducted on the impact of saturated fatty acids on MDD, recent investigations support an impact of this fatty acid subtype on depressive mechanisms. The specific accumulation of palmitic acid in the hypothalamus of mice exposed to a high-fat diet was found to be associated with the onset of depression and the inhibition of the 3′,5′-cyclic AMP (cAMP)/protein kinase (PKA) signaling pathway (140) (see Figure 1). These findings offer a potential explanation for the association between the consumption of a Western-type diet and the prevalence of major depressive disorder (MDD) [(141); also see later].

Finally, the proposition that eCB signaling may be involved in MDD is primarily supported by evidence indicating that the eCB system functions as a sophisticated signaling system that modulates synaptic transmission through retrograde, non-retrograde, and neuron-astrocyte signaling mechanisms (112). The disruption of eCB signaling has been associated with negative emotional states (142) and heightened stress responses (142). Furthermore, there is evidence suggesting that eCB signaling plays a role in the dysregulation of the HPA-axis. For instance, the signaling of amygdalar AEA through CB1 receptors has been shown to contribute to the magnitude of the HPA response in rats (143). In addition to the HPA-axis, the eCB signaling system has also been implicated in neuroinflammation. Specifically, studies have demonstrated that rodents with overexpression of the CB2 receptor exhibit reduced levels of stress-induced inflammatory cytokines, whereas CB2 receptor knockout animals display an exacerbated neuroinflammatory phenotype (144). These observations are likely attributed to the expression of CB2 receptors on microglia, as both associative and causative evidence have linked the CB2 receptor on microglia to anti-inflammatory properties (114), which may be modulated by AEA (145). While the role of the CB1 receptor in neuroinflammation has been investigated in some studies (146), conclusive evidence is lacking, necessitating further research in this area. Thirdly, a substantial body of evidence supports the role of the eCB system in neurogenesis. Specifically, the CB1 receptor has been identified as a mediator of adult neurogenesis (147, 148), a process that is likely facilitated by 2-AG in rodent models (149). In contrast, contradictory findings have been observed regarding the involvement of the CB2 receptor in neurogenesis, particularly in animals lacking CB2 receptors (150, 151). Consequently, further research is necessary to establish definitive conclusions regarding the role of the CB2 receptor in neurogenesis. Lastly, several studies have even claimed the eCB receptors and their ligands as direct antidepressant targets. Indeed, AEA was shown to treat depression induced by acute stress in preclinical models (152). Furthermore, inhibitors for monoacylglycerol lipase (MAGL), the 2-AG-degrading enzyme, were successfully tested in preclinical models of MDD (153) and are likely to enter clinical trials soon.

Together, it is clear that fatty acids play a substantial role in the regulation of mood and as such the development of MDD with a particular role for the diet in maintaining this balance. Nevertheless, although their role and even their modulation appears advantageous to treat MDD, using fatty acids as novel targets for treatment seems premature at this stage as a better understanding of the underlying mechanisms of action seems highly desired given their wide physiological impact on cellular processes. Still, given the exceptional findings that have been generated by the use of this lipid, treatment regimens with fatty acids (in some form) appear to have an auspicious future.

## 3. Depressive signs in lipid-related disorders

Another approach to underscore the impact of lipids in MDD is by highlighting the comorbidities and/or reciprocal relationships between depressive symptoms and lipid-related disorders. For this purpose, the following paragraphs shortly highlight the links between MDD and obesity and type 2 diabetes.

### 3.1. Obesity

Overweight and obesity are generally defined as excessive fat accumulation that pose individuals at risk to health (154), which is often measured by the use of the body mass index [BMI; though the latter metric does not accurately represent fat amount and content (155)]. Excess fat is initially stored in adipose tissue depots that are able to expand in size, concomitantly causing weight gain. However, when the maximal capacity to store fat in the adipose tissue depots has been reached, lipids will accumulate in alternative, more deleterious places such as the arteries and the liver. This latter process is also referred to as ectopic lipid accumulation and is associated to pathophysiological processes such as inflammation (among other processes) (156).

Indeed, the low-grade inflammatory state during obesity is considered a prime instigator of depression onset in obesity (17, 157, 158), even in metabolically healthy obese individuals who are characterized by only minor inflammatory elevations (159). A potential underlying mechanism for the development of a depressed state resulting from persistent low-grade inflammation involves the manifestation of neuroinflammation, which can lead to alterations in brain structure and excitability (17).

Another pathway that links MDD to obesity is via the HPA axis. In stressed individuals, weight gain is attributed to dysregulation of the HPA axis, mediated by cortisol-induced activation of brain glucocorticoid receptors, which subsequently promotes the consumption of palatable food. This effect leads to a reduction in HPA activity, providing temporary alleviation of negative affective states (160). This causal cycle between obesity, stress and depression is congruent with the well-established finding that obese individuals (adults, adolescents and children) are at increased risk to develop depression (161, 162), implying that obesity might also predispose individuals to depression. However, the relationship between obesity and depression seems rather bidirectional as underlying mechanisms are very closely intertwined (see the examples of inflammation and HPA-axis here above) (158, 162). This close connection was further exemplified by evidences showing that deep-brain stimulation in the nucleus accumbens, which is an antidepressive approach (163), is also effective in reducing body weight in obese rodents and patients (164), implying an overlap in the brain structure and neurotransmitter systems controlling mood at one hand and motivation for food at the other hand. As such, depression and obesity appear rather as a reciprocal relationship with each component contributing to and aggravating the other (162).

Indeed, vice versa, MDD patients categorized under the specifier atypical depression strongly associate with elevated BMI and are characterized by a plasma lipid profile which is similar to obese individuals (165). As such, obese individuals tend to develop the atypical MDD subtype (84), which is often associated with a more complex disease course [partly due to reduced sensitivity to pharmacological antidepressants (166)]. An underlying mechanism, which has been linked to the atypical subtype of MDD, is leptin signaling. While leptin is most recognized for its regulatory function on satiety and appetite (167), leptin resistance associates specifically with atypical MDD in humans (168). Furthermore, treatment with metreleptin, a leptin analog, possesses antidepressant properties in patients (169), further supporting the notion that leptin signaling is likely involved in atypical MDD.

### 3.2. Type 2 diabetes

Next, to inflammation, ectopic lipid accumulation also results in the development of insulin resistance in both skeletal muscle and liver tissues (170). Insulin resistance refers to a pathological condition wherein cells exhibit an inadequate response to insulin, leading to disruptions in both lipid and carbohydrate metabolism (171). Diabetes mellitus is a clinical manifestation of insulin resistance and is the most prevalent metabolic disorder in humans, potentially leading to severe complications and organ failure (172). Diabetes mellitus is commonly categorized into three distinct types (type 1, type 2, and gestational), with type 2 diabetes representing the majority of diabetic cases, accounting for approximately 90%–95% of total diagnoses. Type 2 diabetes is typically characterized by a combination of pancreatic beta cell deterioration and dysfunction, alongside insulin resistance in target tissues (173).

The co-occurrence of MDD and type 2 diabetes has gained significant attention among researchers due to the parallel increase in the prevalence of both disorders. As such, investigations have been conducted to explore their potential interrelation. Evidence suggests that depression is associated with a significantly higher risk of developing type 2 diabetes, and conversely, individuals with type 2 diabetes have an increased risk of developing depression, although the latter association is less pronounced (174, 175). These observations support the idea that MDD and type 2 diabetes are intertwined and that their association is bidirectional, implying that a comprehensive approach to the management of these disorders may be beneficial (174–178). Indeed, diabetes medications such as metformin promote antidepressant-like responses by mechanisms that are, thus far, unclear (179, 180). Hence, since insulin resistance exerts its influence on numerous aspects of human metabolism beyond lipid metabolism, the exclusive contribution of lipid-related mechanisms to the association between depression and type 2 diabetes remains uncertain, given the present knowledge. However, given the impact of high-fat diets, obesity, and lipid profiles on depressive outcomes (178), it is reasonable to hypothesize that lipids may play a role in the relationship between depression and type 2 diabetes, to some degree, in patients affected by the latter.

## 4. Conclusion

An increasing amount of data demonstrates compelling evidence for the rise of MDD in global society. Although lipids are central players in neurophysiology and pathology, their potential involvement in the context of MDD has received relatively little attention. The evidence presented in this review emphasizes the potential contribution of lipids in elucidating the complex pathophysiology of MDD and in enhancing the efficacy of existing therapeutics. Furthermore, lipids may help explain the already known underlying mechanisms involved in MDD, such as disturbances in inflammation and dysregulation of the HPA-axis. The available evidence also suggests that an unhealthy lifestyle, particularly a high-fat diet, can significantly impact the development of MDD, an aspect that has been controversial in the literature (181). Therefore, further research is necessary to investigate the potential effect of diet on MDD and existing antidepressive approaches. Finally, the bidirectional association between MDD and lipid-related disorders such as obesity and type 2 diabetes implies the existence of shared underlying mechanisms, which could be employed for the management and treatment of both conditions.

## Author contributions

AH and TH: writing—review and editing. All authors contributed to the article and approved the submitted version.

## Funding

This research was supported by a Kootstra Talent Fellowship for talented postdoctoral researchers (TH) and a ZonMw Off Road grant (number: 04510012010010).

## Conflict of interest

The authors declare that the research was conducted in the absence of any commercial or financial relationships that could be construed as a potential conflict of interest.

## Publisher’s note

All claims expressed in this article are solely those of the authors and do not necessarily represent those of their affiliated organizations, or those of the publisher, the editors and the reviewers. Any product that may be evaluated in this article, or claim that may be made by its manufacturer, is not guaranteed or endorsed by the publisher.
